# A scalable overexpression of a thermostable recombinant poly-histidine tag carboxyl esterase under lambda promoter: purification, characterization, and protein modelling

**DOI:** 10.1186/s43141-023-00610-w

**Published:** 2023-12-12

**Authors:** Nadia A. Soliman, Safaa M. Ali, Mahmoud E. A. Duab, Yasser R. Abdel-Fattah

**Affiliations:** 1 Bioprocess Development Department, Genetic Engineering and Biotechnology Research Institute (GEBRI), City of Scientific Research and Technological Applications (SRTA-City), Universities and Research Institutes Zone, New Borg El-Arab City, Alexandria, 21934 Egypt; 2 Nucleic Acid Research Department, Genetic Engineering and Biotechnology Research Institute (GEBRI), City of Scientific Research and Technological Applications (SRTA-City), Universities and Research Institutes Zone, New Borg El-Arab City, Alexandria, 21934 Egypt

**Keywords:** Affinity chromatography, Bioreactor, Histidine affinity tag, Scaling up, Sub-cloning

## Abstract

**Background:**

As a white biotechnological trend, esterases are thought to be among the most active enzymes’ classes in biocatalysis and synthesis of industrially importance organic compounds. Esterases are used in many applications such as the manufacture of pharmaceuticals, cosmetics, leather, textile, paper, food, dairy products, detergents, and treatment of some environmental pollutants.

**Results:**

A poly-histidine moiety was added to the C-terminal end of the *Geobacillus* sp. gene encoding carboxyl esterase (*EstB*, ac: KJ735452) to facilitate one-step purification. This recombinant protein was successfully expressed in *Escherichia coli* (*E. coli*) under control of Lambda promoter (λ). An open reading frame (ORF) of 1500 bps encoding a polypeptide of 499 amino acid residues and a calculated molecular weight (54.7 kD) was identified as carboxyl-esterase B due to its conserved glycine-X-serine-X-glycine motif (G-X-S-X-G) and its high similarity toward other carboxyl esterases, where the 3-D tertiary structure of EstB was determined based on high homology % (94.8) to Est55. The expression was scaled up using 7-L stirred tank bioreactor, where a maximum yield of enzyme was obtained after 3.5 h with SEA 51.76 U/mg protein. The expressed protein was purified until unity using immobilized metal affinity chromatography (IMAC) charged with cobalt and then characterized. The purified enzyme was most active at pH 8.0 and remarkably stable at pH (8–10). Temperature optimum was recorded at 65 °C, and it kept 70% of its activity after 1-h exposure to 60 °C. The active half-live of enzyme was 25 min at 70 °C and a calculated T melting (Tm) at 70 °C. The determined reaction kinetics Michaelis–Menten constant (*K*_*m*_), maximum velocity rate (*V*_*max*_), the turnover number (*K*_*cat*_), and catalytic efficiency (*K*_*cat*_*/K*_*m*_) of the pure enzyme were found 22.756 mM, 164.47 U/ml (59.6 min^−1^), and (2.619 mol/ min), respectively.

**Conclusion:**

Creation of a recombinant 6 × -His estB derived from a thermophile *Geobacillus* sp. was performed successfully and then overexpressed under *λ*-promoter. In a bench scale bioreactor, the overexpression was grown up, followed by one-step purification and biochemical characterization. The recorded promising pH and temperature stability properties suggest that this expressed carboxyl esterase could be used in many industrial sectors.

## Introduction

Lipolytic enzymes are divided into two groups: carboxyl esterases (EC 3.1.1.1) and the lipases (EC 3.1.1.3). They catalyze the breaking and synthesis of ester bond. Carboxyl esterases differ from lipases in that they do not require interfacial activation and prefer to hydrolyze water-soluble acylglycerols with short-chain lengths (acyl chains < 10 carbon atoms) as substrates [1]. Carboxyl esterases are a type of “real esterase” that can catalyze interesterification, esterification, peracid, and aminolysis production, according to Bornsheuer [1]. Also, lipases can be defined as carboxyl esterases which catalyze the hydrolysis of long-chain acylglycerols to glycerol, free fatty acids, and mono- and diglycerides. Thus, lipolytic enzymes are considered the most frequently employed biocatalysts throughout nature. The optimal environment for an esterase to catalyze the hydrolysis of the bonds in a carboxyl ester made of short-chain fatty acids is aqueous solution. Esterases can also catalyze the synthesis and transesterification of esters in water-free or water-restricted media [2–4]. Esterases are used in a number of industrial applications in the manufacture of pharmaceuticals, cosmetics, textile, paper, leather, animal foods, foodstuffs (products of dairy), detergents, and environmental management. In pharmacology, esterases are used for the creation of crucial chiral chemicals owing to their great region stereospecificity [5, 6]. The widespread use of this enzyme in different sectors of industry has sparked interest in the discovery and characterization of new esterases of microbial origin [7, 8]. Esterases from microorganisms are potent biocatalysts that efficiently hydrolyze many types of polyester, including polylactic acid (PLA), in the process of plastic depolymerization. Some researchers [9, 10] have reported that many metagenomic esterases are able to hydrolyze numerous polyester substrates, including PLA. Because PLA degrades and generates CO_2_, recycling is essential. In this regard, depolymerization of PLA or other polymers and reutilization of monomers for new plastic synthesis are considered an attractive recycling option [11]. Many pharmaceuticals and prodrugs, including clopidogree, aspirin acetylcholinesterase, and irinotecan, as well as antiplatelet medications including imidapril, delapril, and temocapril, are chemically modified in large part by esterases [12, 13].

Enzymes, the agents of life, are generally considered as catalysts, and their nature are proteins. They are protein molecules that carry out functions within a cell; most cells contain thousands of them. They are very efficient catalysts and have wider biotechnological expansion than chemical catalysts.

The prediction of protein structure is mostly dependent on sequence and structural homology [14]. The function of a protein is largely dependent on its 3D structure; hence, protein structure prediction or modelling is very significant. Similarly, a protein’s 3D structure is determined by its amino acid residues. A minor change in the protein sequence can cause significant structural changes in the native structure. However, it is very challenging to determine a protein’s natural structure when it exists in the physiological settings of the body. Accurate information of protein 3D structure is crucial. The major methods for figuring out the structure of proteins and protein–ligand complexes are X-ray crystallography and nuclear magnetic resonance spectroscopy (NMR) [15, 16].

However, because enzymes may be destroyed by subtle changes in temperature or pH, they are often considered a weak link in biochemical reactions. Scientists now realized that heat-loving organisms called thermophiles contain environmentally friendly enzymes that are stable under high temperatures and could be used in place of more dangerous chemicals that have been concocted by modern industry.

Thermostable esterases, which are generated from thermophiles that have adapted to exist at high temperatures, are a promising study topic. Such enzymes have a protein structure that allows them to be highly resistant to both organic and inorganic solvents as well as to lots of denaturing conditions. Recently, many recombinant thermostable carboxyl esterases have been identified, cloned, and overexpressed in *E. coli* such as carboxyl esterase (*EstA*) from *Geobacillus thermoleovorans* YN [17], esterase from *Geobacillus* sp. ZH1 [18], *EstAZ1* from *Geobacillus thermodenitrificans* AZ [19], and *TtEst2* from *Thermogutta terrifontis* [20]. These enzymes are mostly produced in a small scale (at the flask level), while scalable production is infrequent. Due to the scarcity of high-level investigations on esterase synthesis, this research area is extremely valuable and treasured. Additionally, esterases play a crucial role in the biotechnological sector and are used in a wide variety of applications on a global scale.

**Fig. 1:**
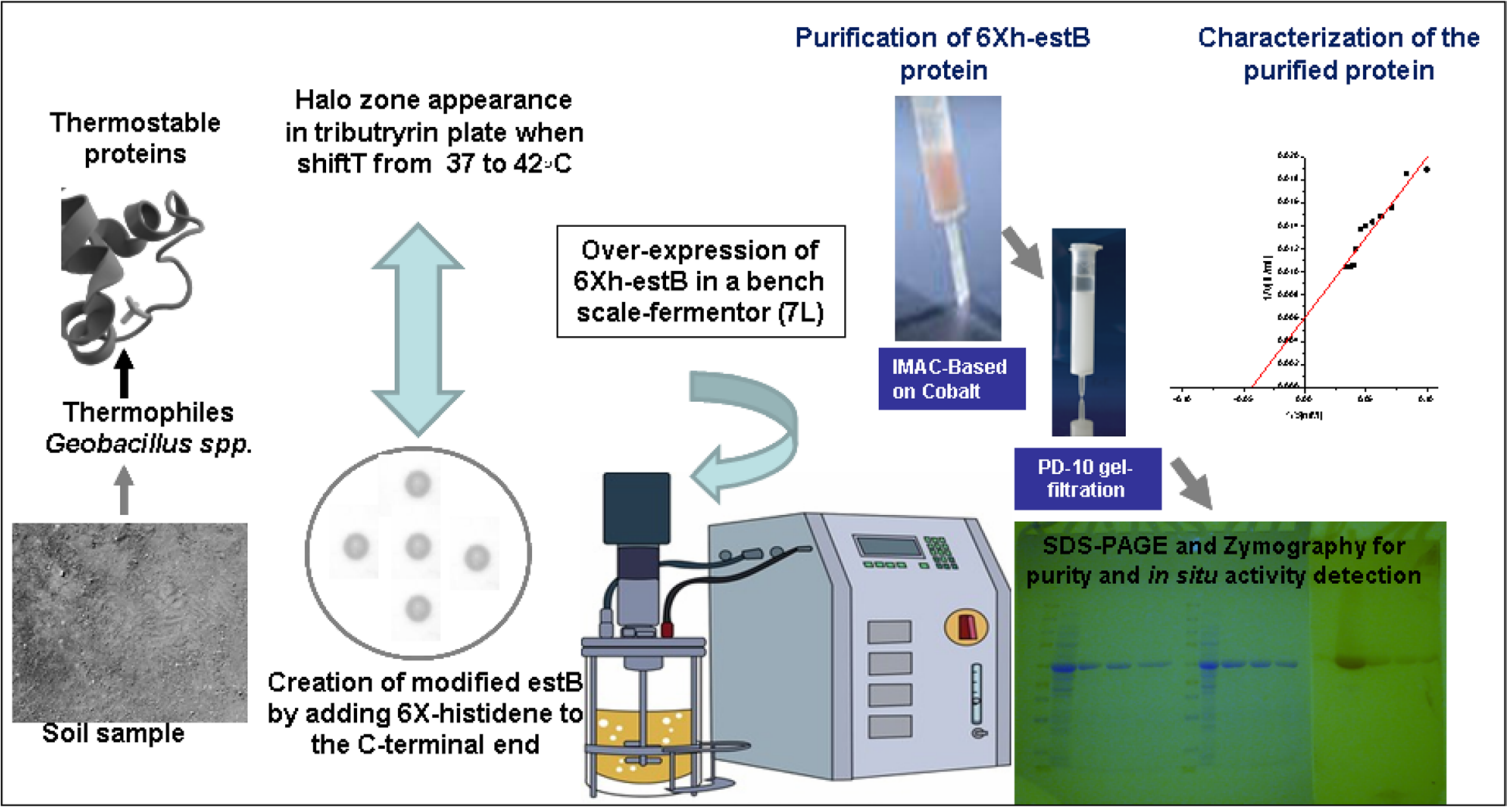
Overall representative scheme of various laboratory experimental steps

The current paper highlighted the overexpression of recombinant 6X His carboxyl-esterase B derived from a thermophile *Geobacillus* sp. isolated from Egyptian desert soil, under temperature shift (*λ*-promoter). A non-costly physical induction protocol was applied to easily scale-up the recombinant protein and cover the uncommon interest in scaling up of esterases. One-step purification and biochemical characterization of the overexpressed protein were carried out as a proactive and preliminary step to boost enzyme visibility. Furthermore, the 3-D tertiary structure was built according to homology sequence approach. This recombinant protein, without a doubt, can be used as a good template for engineering or changing to achieve desired functionality using advanced molecular techniques. Schematic diagram of the laboratory experimental setup is summarized in Fig. 1.

## Materials and methods

### Bacterial strain, plasmids, and growth conditions

*Escherichia coli* DH5 α [F_ endA1 hsdR17 (rk_, mk +) supE44 thi-1λ-gyrA96 relA1 D (argF-laczya) U169] was used for the cloning steps, the propagation of all expression vectors, and expression of recombinant proteins.

Cells were cultivated at 37 °C and 200 rpm in Luria–Bertani (LB) low salt of the following composition (g/L): yeast extract, 5; peptone, 10; and NaCl, 5.0. For selection of plasmids, LB medium was supplemented with 100 mg/L ampicillin. Chemically competent *E. coli* DH5α cells were produced according to Sambrook et al. [21]. pCYTEXP1 plasmid was used for protein overexpression [22].

### Sub-cloning and expression of 6X-Histidine esterase B under λ-promoter

The esterase B gene derived formerly from *Geobacillus* sp. [23] was amplified by polymerase chain reaction (PCR) from the plasmid (pCYTEX-*est*B) using the forward primer (F*Nde*I 5′-*GGAATTCATATG*ATGGAACGAACCGTTGTTGAA-3′) and a tailed poly-histidine reverse primer R-6Xhis *Sal*I: 5′-*GGCCGTCGAC*CTA*ATGGTGATG GTGATGGTG*GCGTCCTTGCCATGCC-3′), in order to fuse a His6-tag to the C-terminal part of the expressed protein to permit one-step purification using affinity chromatography. The restriction sites *Nde*1 and *Sal*I were incorporated into the forward and reverse primer sequence, respectively. The purified, digested (*Nde*I/*EcoR*I) PCR fragment was ligated into the respective sites of pCYTEX-P1 leading to pCYTEX-6X-his *est*B. Transformants were validated by restriction analysis and PCR using the primers SDM1 (5′-CCAACACTACTACGTTTTAACTGAAACAAACTGG-3′) and SDM3 (5′-GCGAACG CCAGCAAGACGTAGCCCAGC-3′. Esterase activities of the transformants were screened using LB_amp_ plates supplemented with tributyrin (0.5%). After induction (temperature shift from 37 to 42 °C), the active clones developed a halo zone.

### Sequence similarity and protein modelling

Sequence similarity investigation was done using BLAST search against sequences available in GenBank (www.ncbi.nlm.nih.gov/blast). Subsequently, the sequence was submitted to GenBank with the accession number KJ735452.

The 3D model structure of estB was determined using SWISS-MODEL server, which relies ProMod3 [24], an internal comparative modelling engine built on OpenStructures [25]. The structure of a thermostable esterase Est55 from *Geobacillus stearothermophilus* (2ogs.1. A) at 10.2210/pdb2OGS/pdb. was selected as template.

### Fermentation experiment

#### Bioreactor

Batch culturing was carried out in a 7-L benchtop bioreactor [Bioflow® & CelliGen® 310, New Brunswick Scientific (USA) Company] with two six-bladed disc-turbine impellers, four baffles, and a digital control unit. The approach was automated using the AFS-BioCommand multiprocess management tool, which is a computer-aided data bioprocessing system. Temperature, agitation, and pH value were set at 37 °C, 200 rpm, and 7.0, respectively. Automatic input of 2N NaOH kept the pH in check. Through a sterile filter, compressed gas was delivered at 1.0 VVM (air volume per broth volume per minute). METTLER TOLEDO electrodes were used to control the pH and measure the dissolved oxygen level and pH values online.

## Experiment

*E. coli* DH5α carrying the plasmids (pCYTEX-*Est*B-6xhis) encoding the esterase gene was grown overnight in 200-ml LB_amp_ medium at 37 °C. A 4 L of LB_amp_ medium was inoculated with 5% overnight culture and cultivated at 37 °C, pH 7.0, and 200 rpm until an OD_600_ of 0.7–1.0. Upon induction by shifting the temperature from 37 to 42 °C, cells were cultivated for additional 4 h, where the activity was recorded every half hour.

### Purification of the recombinant 6Xhis esterase

The pellet was collected at the peak of activity, centrifuged (10 min, 4000 rpm, 4 °C), and sonicated (3 × , 1 min), and the supernatant containing the soluble protein fraction was separated and used for purification.

The recombinant protein was purified under native conditions using TALON, Metal Affinity Resin (BD Biosciences Clontech, Heidelberg, Germany) according to the manufacturer’s guidelines at pH 7.0 with imidazole for elution at room temperature. A column was filled with 8 ml of IMAC matrix and equilibrated with three volumes of saline phosphate buffer (50 mM, pH 7.0). It was rinsed with saline phosphate buffer after applying the sample into column. Fraction-wise elution of the bounded protein was achieved by using 150-mM imidazole in saline phosphate buffer (50 mM, pH 7.0). Gel filtration with a PD-10 (gel-filtration column) was established to remove any remaining imidazole (Amersham Biosciences, Freiburg, Germany).

### Protein determination

Protein concentration was measured at λ_280nm_ (Biochrom-Libra S32PS, England). A standard curve was prepared using L-tryptophan at concentrations (10–100 μg/ml) [26].

### Sodium dodecyl sulfate–polyacrylamide gel electrophoresis (SDS-PAGE) and zymogram

SDS-PAGE was performed on 12% running gels as described by Laemmli [27], and resolved proteins were visualized by Coomassie staining following standard procedures. A wide range of molecular weight pre-stained protein standard (Protein Marker XXL DeLuxe 10–260 kDa) was used as molecular mass marker. The protein samples containing esterase were separated by SDS-PAGE and renatured in 100-mM Tris–HCl buffer, pH 7.5 containing 0.5% Triton X-100 for 4 h at 4 °C. The gel was finally incubated for 5 min at RT in a developing solution which contains 3-mM α-naphthyl acetate, 1-mM Fast Red TR (Sigma), and 100-mM sodium phosphate buffer, pH 7.5. Esterase activity was detected by the appearance of brown-colored bands within the gel.

### Determination of hydrolase activity

Quantitative determination for hydrolytic activity of esterase toward *p*-nitrophenyl-laurate (*p*-NP-) substrate (Sigma-Aldrich Chemie GmbH, Germany) was measured spectrophotometrically according to Becker et al. [28]. The enzymatic reaction was performed using 100-μl substrate solution (25-mM *p*-NP-laurate in absolute ethanol), which was added to appropriately diluted cell lysate (25 μl) dissolved in 725-μl phosphate buffer (50 mM, pH 7.2). A total of 250 μl of 100 mM Na_2_CO_3_ were added after 10 min of incubation at 50 °C, and the mixture was centrifuged at 4 °C (10 min, 13,000 rpm). At 420 nm, the absorbance of liberated *p*-nitrophenol was determined. Under test conditions, one unit (U) of enzyme activity is defined as the quantity of enzyme that liberates 1 μmol of *p*-nitrophenol/min.

### Characterization of the purified recombinant esterase

#### Effect of temperature

The effects of temperature and pH on the enzyme activities were measured using p-NP laurate as substrate. Temperature optimum of enzyme was evaluated by testing a wide range of temperatures (40–85 °C), and the relative activities [%] were measured. The thermostability of the studied enzyme was investigated by measuring the residual activities after 60-min exposure (5-min interval) of the enzyme to temperatures 40, 50, 60, and 70 °C. Tm was calculated from the thermal stability curve according to Saqib and Siddiqui [29]. In Tm, the point corresponds to the temperature when the activity lowers to 50%.

#### Effect of pH

The pH optimum was evaluated by testing different pHs ranging from 5.0 to 10.0. Enzyme reaction at each pH value was carried out at the optimum temperature, and its relative activities [%] were measured. Also, pH stability was tested by incubating the enzymes at pHs 3, 4, 5, 6, 7, 8, 9, and 10 for 24 h, and then the activities were measured and compared.

#### Effect of substrate concentration kinetic

Determination of the kinetic parameters for the hydrolysis of p-NP laurate by the studied recombinant esterase was calculated according to the method of Lineweaver–Burk plot [30], at substrate concentrations which ranged from 10 to 30 mM. Values of *Vmax* (U/ml) and *K*_*m*_ were determined, and all the reactions were carried out at temperature (60 °C) and pH (7.4). Also, both of the turnover number (*k*_*cat*_), and catalytic efficiency (*k*_*cat*_*/k*_*m*_), of the purified enzyme were calculated.

## Results

### Construction of 6xhis recombinant EstB and scaling up the production

**Fig. 2:**
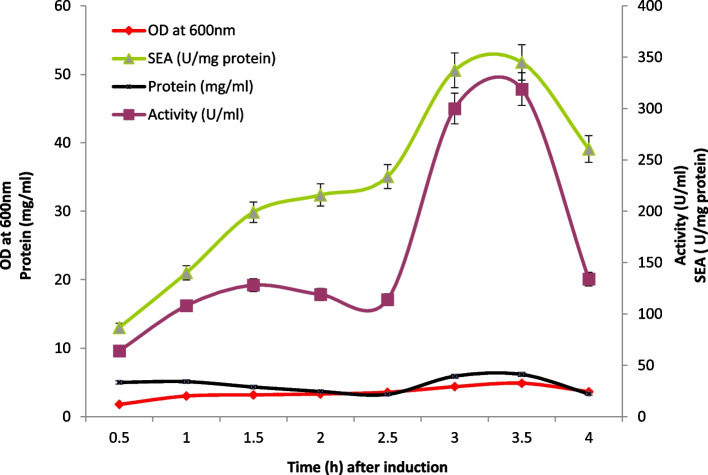
Monitoring of growth, protein, activity, and specific enzyme activity for the recombinant poly histidine carboxyl-esterase B expressed under lambda promoter in 7-L fermentor after induction

The 6X-histidine *Est*B was successfully constructed (pCYTEX-6Xhis *est* B) and then expressed in *E. coli* DH5α host cell under the control of temperature-inducible λ-PL-promoter. The production of recombinant 6Xhis esterase B was scaled up using 7-L bench-top bioreactor. The host cell which carried the gene was allowed to grow in the bioreactor, and then the expression was induced by temperature shift from 37 to 42 °C when optical density reached to 1.0. Samples were withdrawn from sampling gauge at different time intervals and assayed for cell growth (OD_600_nm), protein concentration, and esterase activity by using pNP-laurate as substrate. A typical profile is shown in Fig. 2, where the enzyme (esterase B) production started after stimulation and increased continuously, reaching a maximum after 3.5 h of induction in correlation to the cell growth and SEA (51.7 U/mg protein).

### 3D model structure of the recombinant EstB

**Fig. 3:**
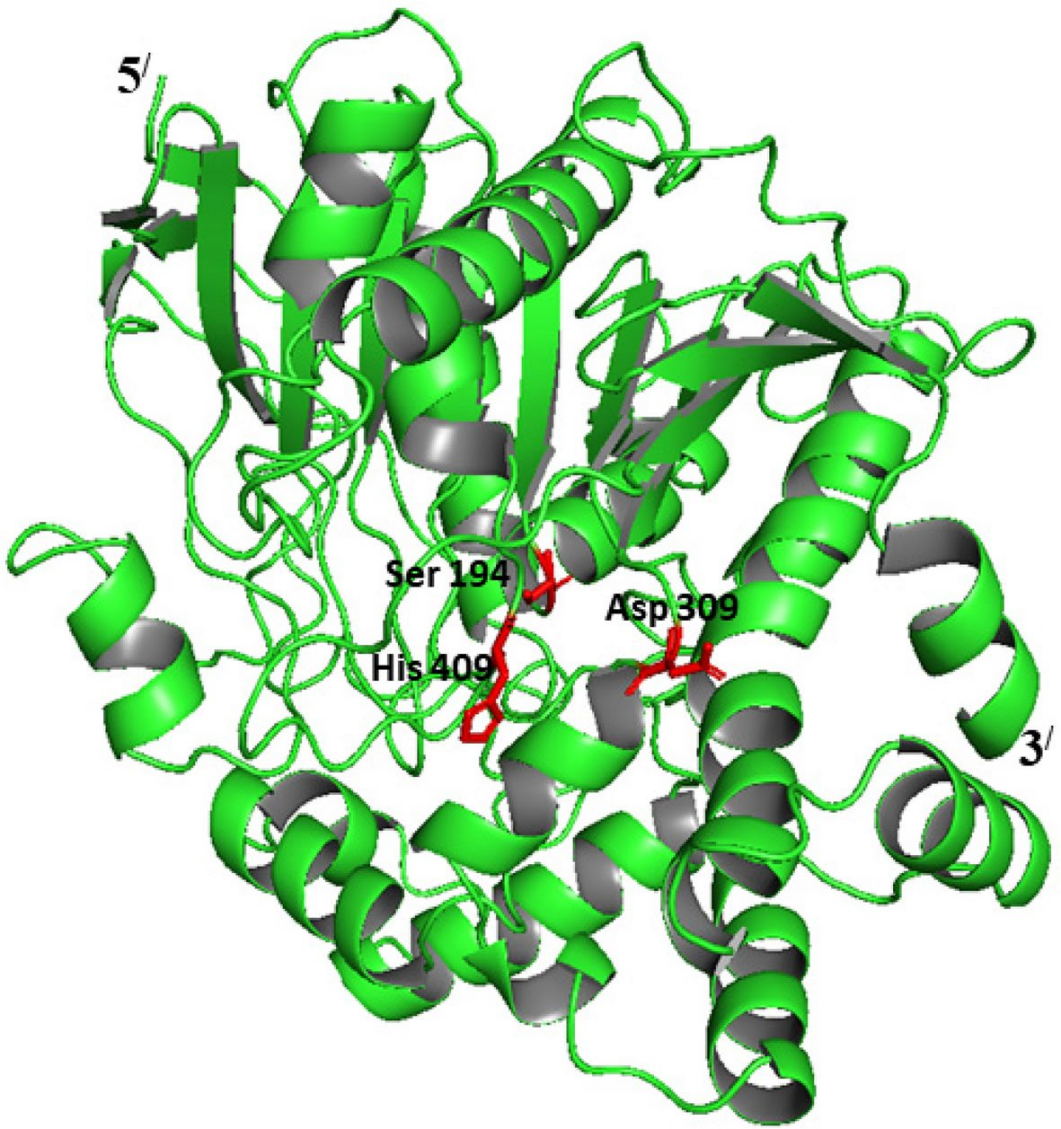
The 3D model structure of estB based on esterase Est55 from *Geobacillus stearothermophilus* (2ogs.1. A) at 10.2210/pdb2OGS/pdb as template, viewing the catalytic triad

The 3D model structure of estB (Fig. 3) was determined by using a thermostable esterase Est55 from *Geobacillus stearothermophilus* (2ogs.1. A) at 10.2210/pdb2OGS/pdb. as template with 94.8% sequence identity. The enzyme keeps a GESAG motif containing an active serine (S) located within a highly conserved catalytic triad of Ser^194^, Asp^309^, and His^409^ residues as shown in Fig. 2.

### Purification of the recombinant EstB

**Fig. 4:**
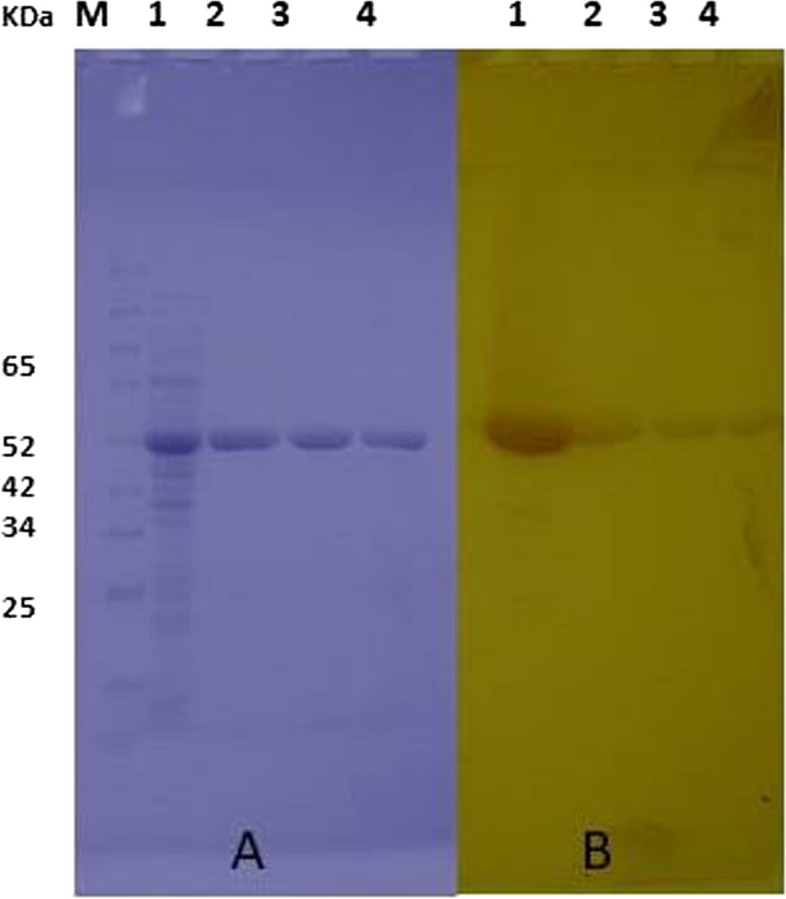
SDS-PAGE analysis of expression of EstB-polyhistidine. **A** Protein detection by staining the gel with Coomassie brilliant blue. **B** In situ activity detection by staining the gel with α-naphthyl acetate and Fast Red TR. Lane 1: crude cell extract of *E. coli* DH5α/pCYTEX-6X his EstB after induction; lanes 2, 3, and 4: the most active purified fraction of EstB-polyhistidine developed after IMAC and gel filtration, respectively

One-step IMAC purification of the esterase (B) led to a nearly homogenous protein of ~ 54 kDa as shown by SDS-PAGE stained with Coomassie brilliant blue (Fig. 4A, lanes 2 & 3) and activity staining using FAST Red TR and α-naphthyl acetate (lanes 2 & 3 in Fig. 4B). SDS-purity check after gel filtration using a PD-10 column confirmed the purity of the previously existed active band (Fig. 4A & B, lane 4). The poly histidine-tagged esterase was finally purified 16.3-fold with a yield of 58.5% and a SEA 3722 U/mg protein.

### Biochemical characterization of the recombinant EstB

**Fig. 5:**
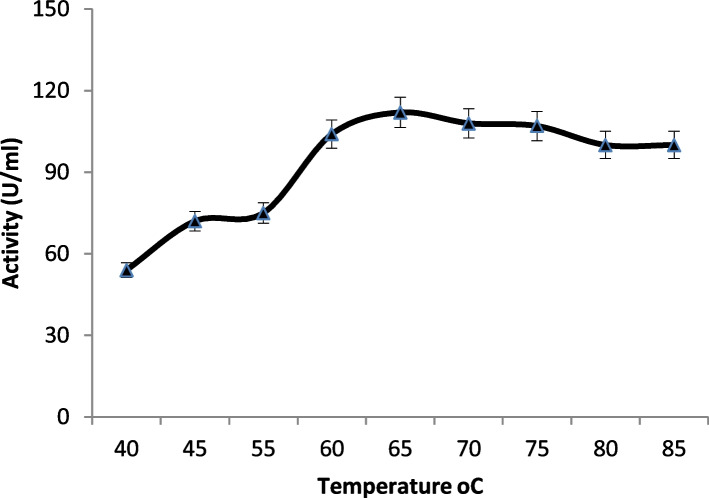
Optimum temperature for the purified recombinant poly-histidine carboxyl-esterase B

**Fig. 6:**
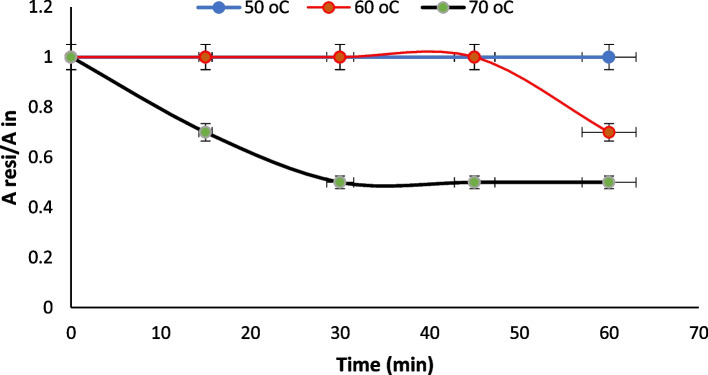
Thermal stability of the purified poly-histidine recombinant carboxyl-esterase B at temperatures 50 °C, 60 °C, and 70 °C as the reduction in the proportion of the residual activity (Aresi) compared with initial activity (Ain) over time

**Fig. 7:**
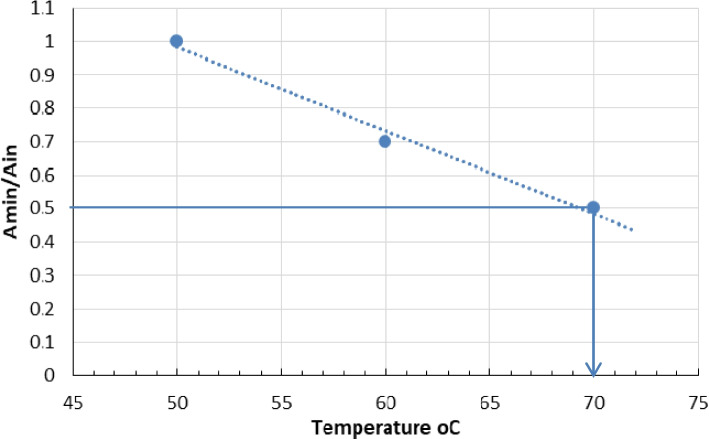
Valuation of melting temperature (Tm) for the purified recombinant 6Xhis estB that corresponds to the temperature when A minimum (Amin)/A initial (Ain) equals 0.5

The effects of temperature and pH on the recombinant esterase (B) activity and stability were tested using p-NP-laurate. As shown in Fig. 5, the esterase is most active at temperatures between 60 and 70 °C, and the optimum was recorded at 65 °C. The esterase B is fully stable for 30 min at 60 °C, but extending the incubation time to 60 min leads to a 30% loss in activity and 50% loss in activity after 25-min exposure to 70 °C as shown in Fig. 6. The calculated Tm (70 °C) was shown in Fig. 7 based on the thermal stability curve. Tm, the point, corresponds to the temperature when the minimum activity (Amin)/initial activity (Ain) drops down to 0.5.

**Fig. 8:**
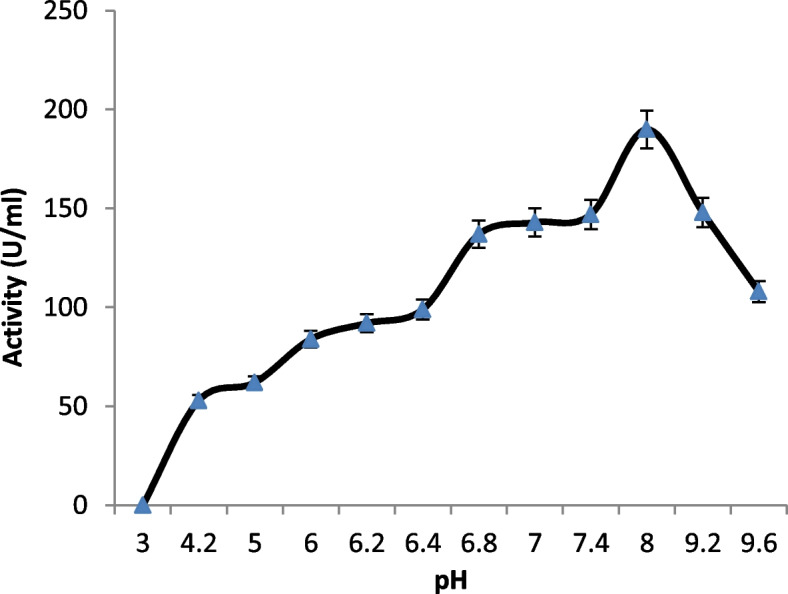
Optimum pH for the purified recombinant poly-histidine carboxyl esterase B

When the recombinant esterase B assayed at various pH values at 65 °C, it showed high activity mainly in alkaline conditions ranged 7.4–9.2 (Fig. 8), and the optimum was recorded at 8.0. The esterase B showed complete stability in a pH range 7–9 for 24 h, whereas 40% drop in stability happened after exposure of the purified enzyme to pH 10 (complete data not shown).

**Fig. 9:**
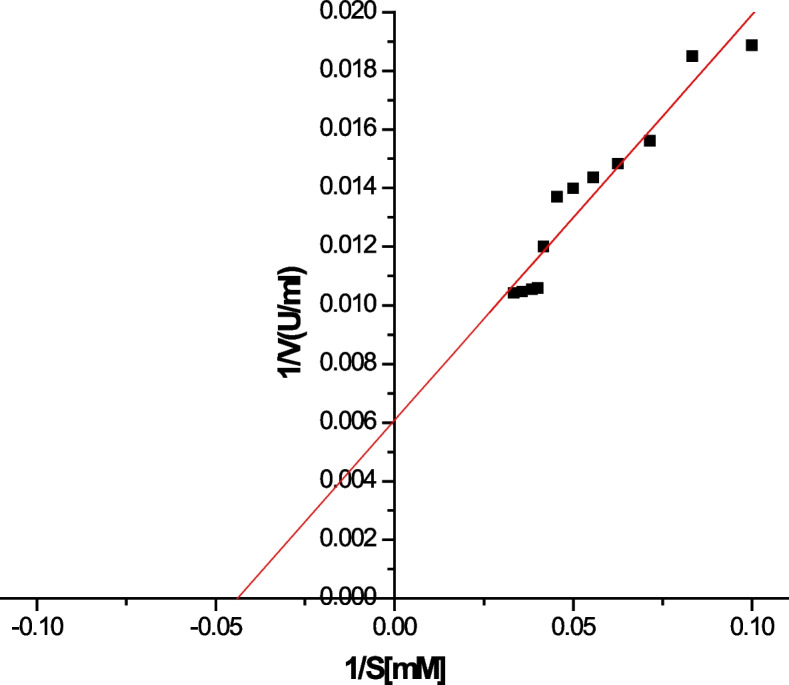
Lineweaver–Burk plot for the purified poly-histidine recombinant carboxyl-esterase B

Kinetic parameters were measured using a spectrophotometric activity assay with p-NP-laurate. The esterase exhibited a simple Michaelis–Menten kinetics for p-NP-laurate. The *Km* value for the conversion of *p*-NP-laurate by the esterase was determined to be 22.756 mM and the respective *Vmax* value was 164.47 U/ml (Fig. 9). Additionally, *kcat* and *kcat/km* were calculated to be 59.6/min and 2.619 mol/min, respectively.

## Discussion

A thermophilic *Geobacillus* isolate from Egyptian desert soil showed extracellular lipase/esterase activities. In order to distinguish between different esterases produced by this strain, cloning strategy was applied, followed by purification of each expressed protein individually [17]. The identification, characterization, and purification of proteins can be accomplished with the help of affinity tags. The antigenic determinant, or area to which an antibody binds, is often a brief sequence of amino acids known as an epitope. Epitope tagging is a process that involves using recombinant DNA to affix a brief sequence, or epitope, to a protein of interest. Epitopes allow for the analysis of protein size, concentration, posttranslational modifications, protein interactions, and intracellular trafficking and, in some cases, provide a method of obtaining pure product (via affinity purification) in order to achieve high-resolution crystallographic resolution. These methods include Western blot analysis, immunoprecipitation (IP), co-immunoprecipitation (co-IP), immunofluorescence (IF), and affinity purification.

To date, a wide range of techniques, such as gene fusion technologies, have been created to facilitate structural and functional research. For instance, improving our knowledge of cell and organism physiology requires clarifying the molecular mechanisms of, for example, the integral membrane proteins’ (IMPs) function and malfunction [31]. This aids in the creation of pharmaceuticals that either suppress or restore protein activity [32]. The genetic engineering of fusion tags, such as brief peptides, protein domains, or entire functional proteins, into target proteins is made possible by the gene fusion approach [33] The resultant recombinant proteins may contain the biochemical properties of the imported fusion tags [34]. As a result, fusion tags can be used to purify and characterize proteins, identify and track protein targets, and improve and evaluate protein expression as well [35].

In this study, we focused on carboxyl-esterase B, and the gene was tailed by addition of 6X-histidine to the C-terminal end to allow the purification of the recombinant protein in one step. The 6X-Histidine stretches were fused successfully, and functional expression of the respective gene was attained under *λ*-promoter. Accordingly, the production was scaled up in 7-L fermentor, where a maximum yield reached after 3.5-h induction (51.7 U/mg protein). Similarly, Soliman et al. [19] scaled up the production of esterase A from *Geobacillus thermodenitrificans* AZ1 in a bioreactor and obtained 22.19 U/min/ml after 3-h induction in LB medium.

The studied recombinant protein of *estB* gene was purified until unity (purification fold 16 times, 58% yield & SEA 3722 U/mg protein) using IMAC in one-step purification. The cell lysate fraction was applied for purification and analyzed by SDS-PAGE and activity stain. SDS and zymography were used to confirm the fractions’ purity. Activity staining of the purified fractions revealed one active band with the expected molecular weights ~ 54 kDa.

As reported by Sood et al. [36], carboxyl esterases are mostly found in a monomer and oligomers of same subunits with molecular weight broadly ranging from 25 to 85 kDa.

Sarkar et al. [37] succeeded in overexpression of *estM2* gene under T7-RNA polymerase, where the His-tagged recombinant protein was purified from cell-free extract of *E. coli* BL21 cells, harboring pETESTM2. Detection of protein purity via SDS showed a single band on PAGE—page with a molecular mass of around 43 kDa. Confirming the functional role of the studied esterase *estB* with α-naphthyl acetate and Fast Red in polyacrylamide gel revealed an esterase-active band at molecular mass ~ 54 kDa. Similarly, Sakar et al. applied the activity staining to fix the molecular mass of the active band of EstM2; they recognized an active band at 43 kDa of the purified native protein (EstM2) [37].

The 6X his purified estB worked optimally at 65 °C and pH 8.0. In addition, estB showed high thermal stability up to 60 °C for 30 min and active in a wide range of pHs 7.0–9.2 up to 24-h exposure, while 30% and 40% loss of activities happened by extending the exposure time to 60 min at 60 °C and 24 h at pH 10, respectively. The half-live of enzyme was recorded after 25 min at 70 °C, and Tm was calculated from thermal stability Fig. (70 °C).

Park et al. [38] demonstrated that EstCS1 is normally stable up to 60 °C with optimal activity at 50 °C. In addition, an optimal activity was observed at pH 8.0, and the EstCS1 possessed its stability within the pH range between 5 and 10.

At 60 °C, both Est30 and Est55 remained stable for more than 2 h; however, at 70 °C, their half-lives were measured after 40 and 180 min, respectively [39].

Protein structure prediction will aid in identifying the structure–function relationship of proteins as well as in investigating and developing protein interactions, which will be extremely useful in the postgenomic era. In this regard, kknowledge-based techniques, notably homology modelling, were used to establish the 3-D structure of the investigated gene. In situations where a query protein shares sequence similarities with a protein whose atomic structure is known, homology modelling is currently the most effective technique for predicting the tertiary structure of proteins [40]. Thus, structure-based alignment between the query protein, namely estB and the sequence with known three dimension (Est55), with 94.8% identity was applied for building the tertiary model structure of estB.

It has been found that many factors imply the main basics of enzymes thermal stability, for example, the occurrence of hydrophobic residues, salt bridges, and low amount of bulky polar amino acids. Low volume of hydrophobic amino acids such as V, G, and A has been found in 85% of known thermostable enzymes [41].

Analyzing the amino acid composition of est B revealed a significant ratio of Ala, Gly, and Val (11.2%, 10.2%, and 6.6%, respectively), which contributed in enzyme thermal rigidity. In further investigation of the amino acid sequence of the studied protein, it was found cysteine residues constitute the least % (0.4), and an increase of Gly, Ser, and Ala pair motif, suggesting a preference for the packing of small residues. The packing plays an important role in thermal adaptation as reported by Meruelo et al. [42].

Thermo and pH stabilities are considered highly selective, major determinants, and prominent features for industrial enzymes. These are highly impacted, and researchers directed to engineered in order to obtain more feasibility for such enzymes [43, 44]. Additionally, to complete enzyme inspection, it is necessary to understand the enzyme reaction kinetics. *V*_*max*_, *K*_*m*_, *k*_*cal*_, and *k*_*cal*_*/k*_*m*_ are useful enzyme kinetic constants that help to understand the practicability and the enzymatic reaction fate. Having a basic understanding of the reaction kinetics further helps control or manipulates the reaction for an efficient reaction process and a higher yield. Therefore, EstB reaction kinetic parameters were determined (*K*_*m*_ = 22.756 mM, *V*_*max*_ = 164.47 U/ml, *K*_*cal*_ = 59.6/min, and *K*_*cal*_*/K*_*m*_ = 2.619 mol/min) according to a Lineweaver–Burk plot; this explained the high affinity of EstB to the tested substrate (*p*-NP-Laurate).

## Conclusion

This study has shed light on some of the industrially important properties such as the hydrogen and thermal stabilities of the carboxyl-esterase B isolated from a thermophile *Geobacillus* sp. Throughout this research, a poly histidine *estB − *gene was accomplished by adding 6X-Histidine moieties to the C-terminal end in order to facilitate the purification by affinity technique in one step. The expressed recombinant protein under *λ*-promoter was scaled up in a benchtop stirred tank bioreactor with maximal SEA (51.7 U/mg protein) after 3.5 h of induction (temperature shift from 37 to 42 °C). Purification until unity for the expressed recombinant protein (6Xhis-estB) was attained at molecular mass ~ 54 kDa, purification fold 16.5, yield % 58.5, and SEA 3722 U/mg. The isolated purified enzyme had the highest activity at 65 °C and pH 8.0, and it was entirely stable at 60 °C for 30 min and pHs 7–9 for 24 h. However, it lost 30% and 50% of activities when the period was extended to 1 h at 60 °C and 25 min at 70 °C. The enzyme lost 40% of activity by exposure to pH 10 for 24 h. Furthermore, EstB has a strong affinity for p-NP-Laurate, with (*K*_*m*_ = 22.756 mM, *V*_*max*_ = 164.47 U/ml, *K*_*cal*_ = 59.6/min, and *K*_*cal*_*/K*_*m*_ = 2.619 mol/min) as determined by a Lineweaver–Burk plot.

## Data Availability

Data will be made available on reasonable request.

## Abbreviations

*E. coli*: *Escherichia coli*
*λ*: Lambda promoter
ORF: Open reading frame
SEA: Specific enzyme activity
*Km*: Michaelis-Menten constant
*Vmax*: Maximal velocity rate
p-NP-: P-Nitrophenyl
EstB: Esterase B
kDa: Kilodalton
h: Hour
IMAC: *Immobilized metal affinity chromatography*
SDS-PAGE: Sodium dodecyl sulfate–polyacrylamide gel electrophoresis
LB: Luria Bertani
G-X-S-X-G: Glycine-X-serine-X-glycine
PLA: Polylactic acid
IP: Immunoprecipitation
co-IP: Co-immunoprecipitation
IF: Immunofluorescence
PCR: Polymerase chain reaction
DNA: Deoxyribonucleic acid
